# Recent Progress in the Engineering of Polymeric Membranes for CO_2_ Capture from Flue Gas

**DOI:** 10.3390/membranes10110365

**Published:** 2020-11-23

**Authors:** Yang Han, Yutong Yang, W. S. Winston Ho

**Affiliations:** 1 William G. Lowrie Department of Chemical and Biomolecular Engineering, The Ohio State University, 151 West Woodruff Avenue, Columbus, OH 43210-1350, USA; han.779@osu.edu (Y.H.); yang.4249@osu.edu (Y.Y.); 2 Department of Materials Science and Engineering, The Ohio State University, 2041 College Road, Columbus, OH 43210-1178, USA

**Keywords:** polymeric membrane, carbon capture, scale up, module, process

## Abstract

CO_2_ capture from coal- or natural gas-derived flue gas has been widely considered as the next opportunity for the large-scale deployment of gas separation membranes. Despite the tremendous progress made in the synthesis of polymeric membranes with high CO_2_/N_2_ separation performance, only a few membrane technologies were advanced to the bench-scale study or above from a highly idealized laboratory setting. Therefore, the recent progress in polymeric membranes is reviewed in the perspectives of capture system energetics, process synthesis, membrane scale-up, modular fabrication, and field tests. These engineering considerations can provide a holistic approach to better guide membrane research and accelerate the commercialization of gas separation membranes for post-combustion carbon capture.

## 1. Introduction

Due to the high energy density, low cost, and the maturity of technologies for energy production, fossil fuels currently account for about 80% of the energy production and 60% of the net electricity generation in the United States [1]. The combustion of fossil fuels produces CO_2_, a greenhouse gas (GHG) that is generally recognized as the main cause for global climate change [2,3,4]. In 2019, the total CO_2_ emissions of the electric power sector in the United States were approximately 1616 million metric tons, within which the coal- and natural gas-fired power plants account for 60% and 39% of the total emissions, respectively [1]. Therefore, the capture and utilization of CO_2_ could not only mitigate the concerns associated with global warming, but could also incentivize the end-use of CO_2_ in the energy sector.

A promising approach to reduce the carbon emissions is post-combustion carbon capture. Under this concept, CO_2_ is captured at large stationary sources, such as power plants, with high purity (e.g., >95%) followed by the sequestration in geological formations (e.g., depleted oil fields and saline formations) or utilization (e.g., enhanced oil recovery and conversion into commodity chemicals) [5]. However, an inherent challenge in post-combustion carbon capture is the low CO_2_ concentration. Due to the use of air for combustion, flue gases are typically discharged at atmospheric pressure with 11–15% CO_2_ for coal-fired power plants and 4–8% for natural gas-fired power plants [6]. The limited thermodynamic driving force for CO_2_ separation imposes a great challenge to develop cost-effective capture technologies.

**Figure 1:**
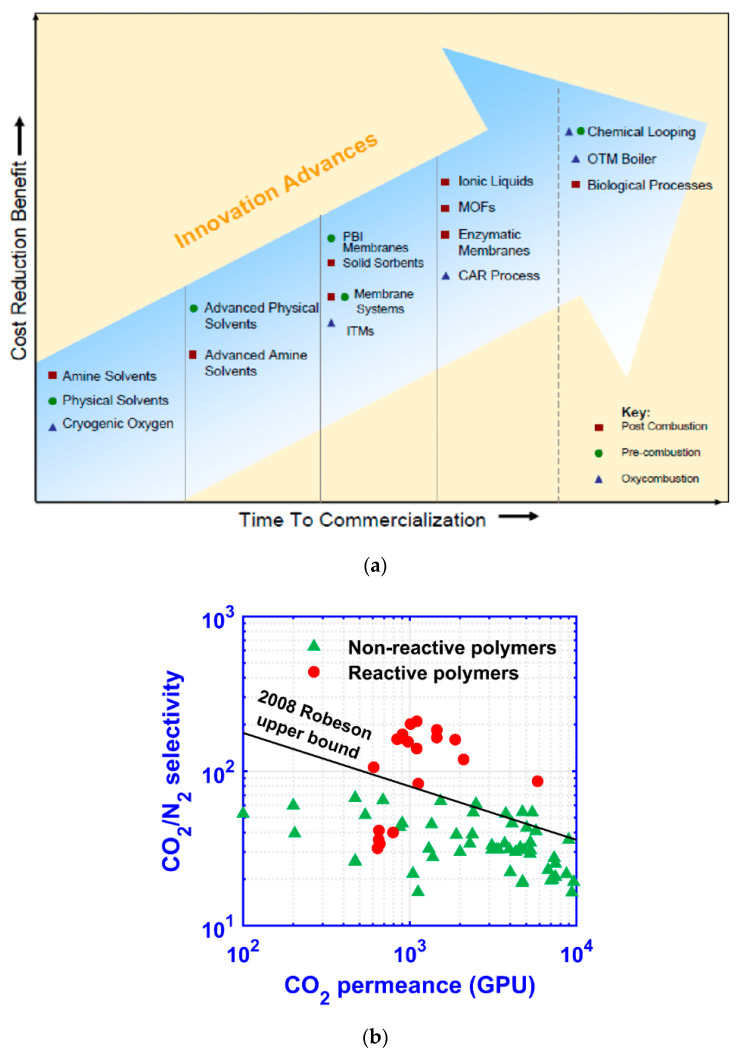
(**a**) Cost reduction benefits vs. time to commercialization for technologies in the U.S. Department of Energy’s Carbon Capture Program (PBI = polybenzimidazole; ITM = ion transport membrane; MOF = metal–organic framework; CAR = ceramic auto thermal recovery; OTM = oxygen transport membrane); Reproduced with permission from [7]. Copyright Elsevier, 2008. (**b**) Transport properties of selected reactive and non-reactive polymers from Refs. [9,10] vs. 2008 Robeson upper bound [12] assuming a membrane thickness of 100 nm.

The urgent need for breakthrough technologies has catalyzed worldwide interdisciplinary research efforts. For instance, the U.S. Department of Energy (DOE) has invested heavily in finding low-cost solutions for post-combustion carbon capture with an aggressive goal to have transformational technologies ready for large-scale demonstration by 2030 [7,8]. Figure 1a shows the cost reduction potential vs. the time to commercialization for technologies in DOE’s Carbon Capture Program, which characterizes membrane-based separation, which is the focus of this review, as a promising technology with high cost reduction benefits and moderate technology readiness for commercialization. Collective efforts from academia and industry have led to the development of novel membrane materials. Fundamental insights into the transport phenomena in these membrane materials have enabled the rational design of transformational membranes with CO_2_ permeances greater than 1000 GPU (1 GPU = 10^−6^ cm^3^ (STP) cm^−2^ s^−1^ cmHg^−1^) and appreciable CO_2_/N_2_ selectivities [9,10,11] as shown in Figure 1b.

In order to close the gap between the membrane material synthesis in a lab-scale setting and the large-scale deployment in the field, however, a scalable fabrication of the membrane must be demonstrated, and the effective membrane process should be designed in accordance with the membrane performance and separation specifications. In addition, the membrane material needs to allow for a multiyear operation in the presence of flue gas contaminants, such as SO*_x_*, NO*_x_*, Hg, and particulate matter. The mass and momentum transfer in the membrane module should also be studied to guide the modular operation. Eventually, the membrane module and membrane process must be tested with actual flue gas to gain information on pretreatment, process dynamics, reliability of non-membrane components (e.g., rotating equipment), etc.

Though important, the abovementioned engineering considerations are largely inadequate in the membrane field. Only a few research institutes or companies were able to advance their membrane technologies to the bench scale or above through integrated programs with fundamental studies, applied research, process synthesis, and techno-economic analysis. Therefore, recent progress in polymeric membranes for post-combustion carbon capture will be discussed in this review from the engineering perspective, including capture system energetics, process synthesis, membrane scale-up, modular fabrication, and learnings from reported field tests. The final goal is to encourage a holistic approach in membrane research and point to the challenges that membrane developers will need to face in field trial and demonstration.

## 2. Membrane Process

### 2.1. Minimum Energy for Separation 

Membrane separation is a pressure-driven process, and the process economics depends on the investment in fixed equipment and the parasitic energy extracted from the power plant to provide the transmembrane driving force. The capital cost is directly correlated with the system footprint, and hence inversely related to the CO_2_ permeance of the membrane. The energy penalty, however, is mainly determined by the membrane selectivity and the process design. In order to understand the energy demand for post-combustion carbon capture and the importance of the membrane selectivity, the minimum work for separation is discussed in this section. 

**Figure 2:**
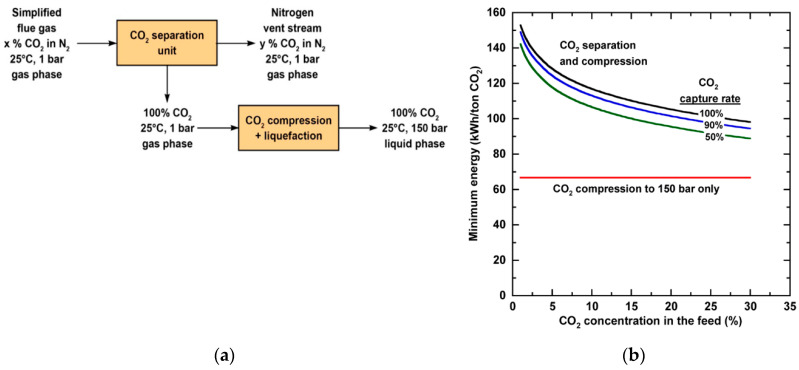
(**a**) Simplified process schematic of CO_2_ separation and compression/liquefaction for a simplified flue gas mixture of CO_2_ and N_2_; (**b**) Minimum energy per metric ton of CO_2_ captured as a function of CO_2_ concentration in a mixture of CO_2_ and N_2_. Reproduced with permission from [14]. Copyright American Chemical Society (ACS), 2012.

For an ideal reversible process, the minimum work for separation is the difference in the Gibbs free energy of the streams entering and leaving the process [13]. Several researchers have investigated the minimum work to separate CO_2_ from flue gas based on a simplified process as shown in Figure 2a [13,14,15]. The CO_2_ concentration fed to the process is determined by the source of flue gas, and the composition of the nitrogen vent stream relies on the CO_2_ capture rate. As shown in Figure 2b, the minimum work to compress and liquefy CO_2_, on a per ton CO_2_ basis, remains constant for the condition specified [14]. The minimum work for separation, however, increases with decreasing feed CO_2_ concentration or increasing capture rate. For a U.S. coal-fired power plant with an average CO_2_ concentration of 13% in the flue gas, capturing 90% of the CO_2_ requires a minimum work of 42.1 kWh/ton. A more demanding minimum work of 62.6 kWh/ton is needed for 90% carbon capture from the natural gas-derived flue gas with 4% CO_2_.

If the work for 90% CO_2_ separation is sourced from the electricity generated from the same coal-fired power plant, the minimum parasitic energy consumes 4.22% of the plant net generation [16]. It should be noted that no practical separation process can operate with the minimum work since it requires an infinitely large system footprint. In reality, separation processes typically render second-law efficiencies, defined as the ratio of minimum to actual energy consumption, in the range of 5–40% [17]. Therefore, the actual energy consumption for a membrane process is at least ca. 10% of the power plant output. It is difficult to analyze the second-law efficiency of a membrane process purely based on first principles. However, this efficiency is typically inversely related to the membrane selectivity, since a lower selectivity allows for more N_2_ permeation through the membrane, meaning that more lost work that cannot be used to extract any free energy. Combining the high minimum work for separation and the limited second-law efficiency, it is paramount to develop highly CO_2_-selective membranes and membrane processes for post-combustion carbon capture.

### 2.2. Process Synthesis 

#### 2.2.1. Single-Stage Process

The goal of process synthesis is to design a membrane architecture to meet the separation specifications. For given membrane properties, the operating conditions are optimized so that the capture cost and parasitic energy are minimized. The thermodynamics exercise in Section 2.1 suggests that there is a trade-off between the separation work and equipment cost. An optimization to reduce the parasitic energy tends to increase the second-law efficiency, while an optimization towards capture cost tends to decrease the second-law efficiency. This optimization problem is further complicated by the stringent separation specifications for post-combustion carbon capture, which require a >95% pure CO_2_ to be produced at a capture rate of 50–90%. As a pressure-driven process, the membrane is best suited for bulk separation, and a separation factor greater than 100 is rare for commercial membranes and processes [18]. 

**Figure 3:**
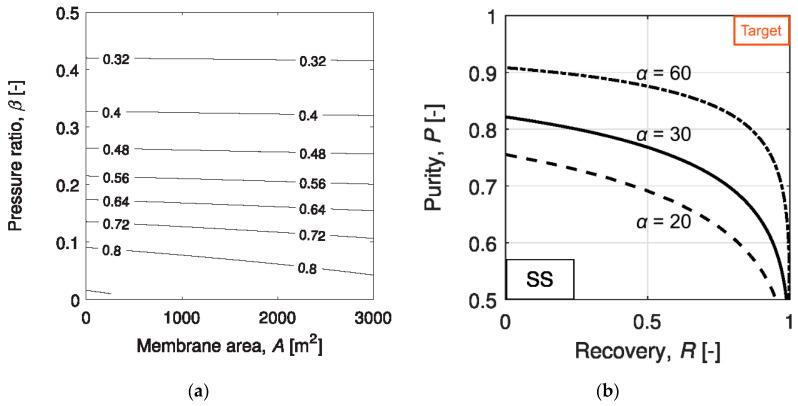
(**a**) CO_2_ purity map at different membrane areas and permeate-to-feed pressure ratios of a single-stage membrane process with a CO_2_/N_2_ selectivity of 50; (**b**) Attainable CO_2_ recovery and purity for a single-stage membrane process with CO_2_/N_2_ selectivities of 20–60. Reproduced with permission from [21]. Copyright Elsevier, 2017.

The demanding separation requirement is best exemplified by various studies on the single-stage membrane process, where the flue gas is separated into a CO_2_-lean retentate and a CO_2_-rich permeate [19,20]. Gabrielli et al. studied the attainable CO_2_ recovery and purity for a single-stage membrane process with different permeate-to-feed pressure ratios and membrane selectivities [21]. As shown in Figure 3a, the CO_2_ purity increases with decreasing permeate-to-feed pressure ratio, indicating a high purity product stream can only be obtained with a large transmembrane driving force. However, even at a permeate-to-feed pressure ratio of 0.1, the product purity is less than 80% for a membrane with a CO_2_/N_2_ selectivity of 50. Further reducing the pressure ratio could improve the enrichment factor but at the expense of process economics. Therefore, a 95% CO_2_ purity can only be achieved by increasing the membrane selectivity or reducing the CO_2_ recovery.

The trade-off between the CO_2_ recovery and purity was also studied by Gabrielli et al. for a single-stage membrane process [21]. As seen in Figure 3b, the purity-recovery Pareto fronts were generated for CO_2_/N_2_ selectivities in the range of 20–60 and permeate-to-feed pressure ratios between 0.01 and 1. Even at a low CO_2_ recovery of 50%, a CO_2_ purity greater than 95% is unattainable with a CO_2_/N_2_ selectivity of 60. Such a demanding selectivity is beyond the capability of most polymeric membrane materials as surveyed in Figure 1b, except for a few reactive polymers relying on facilitated transport. Similar studies were also conducted by Zhai and Rubin [22] and Khalilpour et al. [23] with CO_2_/N_2_ selectivities up to 200. Their results suggest that a CO_2_/N_2_ selectivity > 200 and a permeate-to-feed pressure ratio < 0.05 are required for the 95% CO_2_ purity; however, the CO_2_ recovery must be restricted below 50%. In all, a single-stage process might be used for partial carbon capture provided with the availability of a highly CO_2_-selective membrane. The economics of such a process, however, has not been seen in the literature.

#### 2.2.2. Multi-Stage Processes

**Figure 4:**
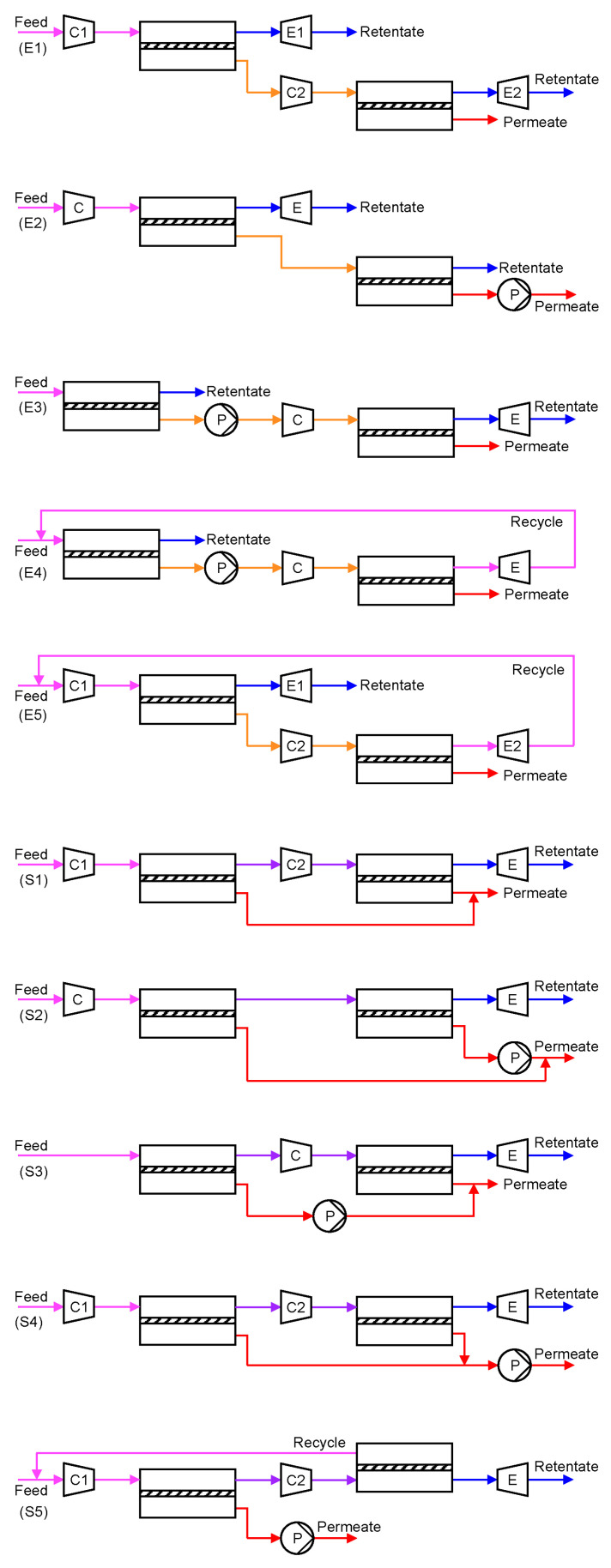
Two-stage membrane processes: (**E1**–**E5**) enriching cascades; (**S1**–**S5**) stripping cascades. Keys: 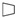 = compressor; 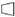 = expander; 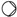 = vacuum pump. Stream: warmer color (e.g., red) = higher CO_2_ concentration; colder color (e.g., blue) = lower CO_2_ concentration.

Limited by the practical transmembrane pressure ratio and the membrane selectivity, a single-stage membrane process cannot achieve a high degree of CO_2_ removal while remaining a purity of >95%. In order to tackle the stringent separation goal and balance the system footprint and energy consumption, various two-stage processes have been designed. Based on the configurations, two general structures can be distinguished: (1) enriching cascade with the permeate of the first stage fed to the second stage and (2) stripping cascade with the retentate of the first stage fed to the second stage. Infinite two-stage processes can be devised based on these two basic cascades by rearranging the rotating equipment. Figure 4 summarizes some of the common designs (E1–E5 as enriching cascades [22,24,25,26,27]; S1–S5 as stripping cascades [28,29,30]). 

In order to achieve a 90% CO_2_ recovery, an enriching cascade requires the first membrane stage to remove ≥90% of the CO_2_ in the flue gas. Recalling the purity-recovery trade-off as shown in Figure 3b, the first enrichment can, at best, render ca. 50–60% CO_2_ in the permeate, which is further enriched by the second membrane stage to achieve >95% CO_2_ purity. The high CO_2_ concentration fed to the second membrane stage provides a higher transmembrane driving force but also leads to a higher stage cut. Therefore, the enriching cascade configuration is typically more energy efficient but with a larger total membrane area [31,32]. On the contrary, the two membrane stages in a stripping cascade configuration each remove a portion of the CO_2_ from the flue gas and produce a permeate stream of >95% purity. However, the low CO_2_ concentration fed to the second stripping stage typically requires a large feed-to-permeate pressure ratio in order to achieve the purity. This results in an overall smaller membrane area but a high parasitic energy consumption [31]. 

In both configurations, pulling a vacuum on the permeate side is generally preferable to feed compression. This is because of the smaller flow rate of the permeate (CO_2_-rich) compared to that of the flue gas (N_2_-rich), albeit the lower efficiency and the larger footprint of a vacuum pump than those of a compressor [20]. In addition, current industrial vacuum pumps can only provide a practical vacuum down to 0.2 atm [10], in which case a mild feed compression should also be considered to further enhance the transmembrane driving force. The choice of the rotating equipment eventually depends on the trade-off between the capital and operational expenditures of the membrane process. 

**Figure 5:**
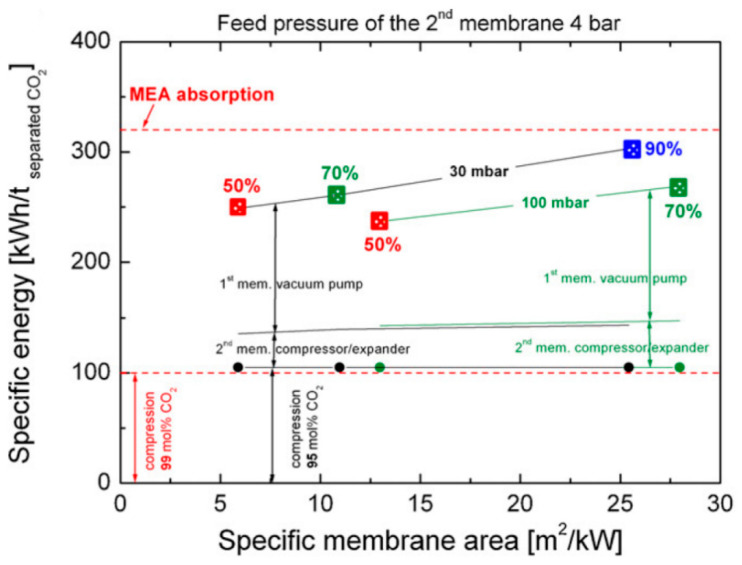
Comparison of parasitic energy consumptions of Process E4 in Figure 4 vs. monoethanolamine (MEA) absorption for CO_2_ removal rates of 50%, 70%, and 90%. A CO_2_ permeance of 185 GPU and a CO_2_/N_2_ selectivity of 43 were used for the membrane. Reproduced with permission from [31]. Copyright Elsevier, 2010.

Of special interest are Processes E4 and E5 in Figure 4, where the retentate of the second enriching stage is recycled back to the feed of the first enriching stage. Because of the closed-loop recycling, the second enriching stage only needs to remove the CO_2_ from 50–60% down to ca. 13% (i.e., the CO_2_ concentration in the flue gas), which reduces its stage cut and membrane area. Zhao et al. studied Process E4 for a commercial membrane with a CO_2_ permeance of 185 GPU and a CO_2_/N_2_ selectivity of 43 [31]. The feed pressure of the second enriching stage was set to 4 bar while the permeate vacuum of the first stage was varied to remove 50–90% of the CO_2_ from a flue gas containing 13.5% CO_2_. As shown in Figure 5, the two-stage enriching cascade can achieve 90% CO_2_ recovery with 95% CO_2_ purity. This study also compared the membrane process with the monoethanolamine (MEA) absorption. Even at 90% CO_2_ recovery, the membrane process exhibits a lower parasitic energy than that of the baseline MEA absorption. Specifically, the vacuum pump and the compression of the 95% CO_2_ account for the major energy consumptions, while the energy penalty caused by the feed compression is relatively small.

**Figure 6:**
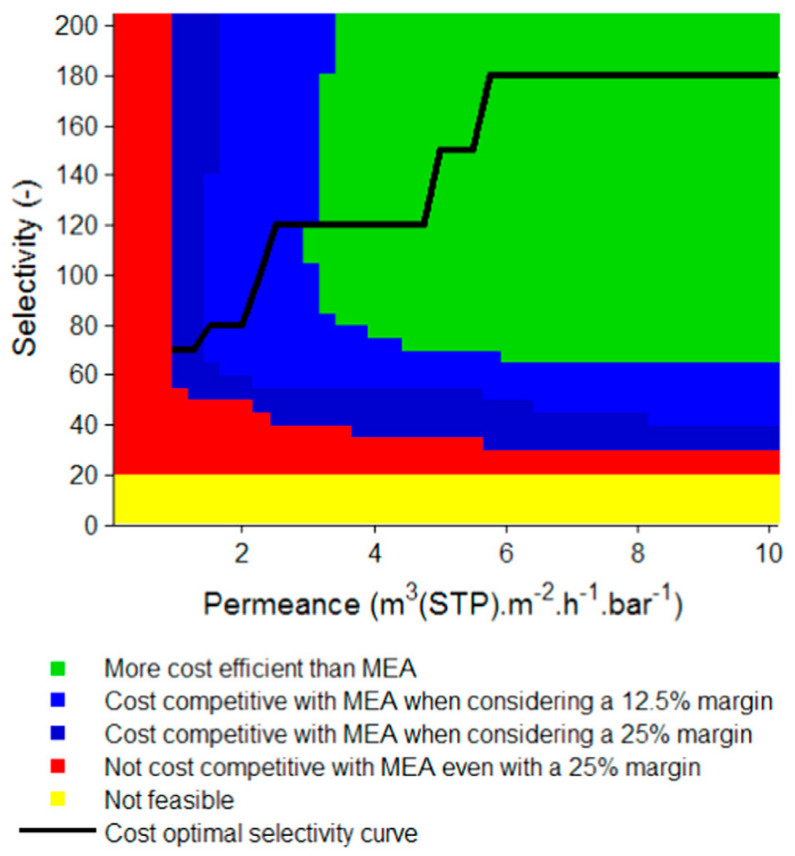
Membrane properties required for Process E4 in Figure 4 to be cost-competitive vs. MEA absorption. Reproduced with permission from [33]. Copyright Elsevier, 2016.

The effect of membrane performance on Process E4 has also been widely investigated. Roussanaly et al. optimized the process performance for CO_2_ permeances ranging from 0–3500 GPU and CO_2_/N_2_ selectivities ranging from 0–200 [33]. As shown in Figure 6, the relative cost efficiencies of the membrane process compared to the MEA absorption are graphically represented for different combinations of membrane permeance and selectivity. The green region represents the range of membrane properties that is definitively cheaper than the MEA-based capture with a margin greater than 25%. Clearly, a selectivity higher than 60 in combination with a CO_2_ permeance higher than 1000 GPU is required for the membrane to be competitive. The black line in Figure 6 corresponds to the optimal selectivity for a given permeance. For advanced membranes with a permeance greater than 1500 GPU (ca. 4 m^3^ (STP) m^−2^ h^−1^ bar^−1^ in Figure 6), a selectivity higher than 120 is required. Once again, this threshold is beyond the capability of most polymeric materials except the facilitated transport membranes. 

In addition, a selectivity higher than 180 is not beneficial for the process economics. In this case, an increase in selectivity leads to a more CO_2_-rich permeate, which requires a larger feed compression or permeate vacuum to maintain the CO_2_ flux [19]. In all, this study suggests that a more permeable membrane needs to be accompanied by a higher CO_2_/N_2_ selectivity in order to fully capitalize on the benefit of the improved permeance.

**Figure 7:**
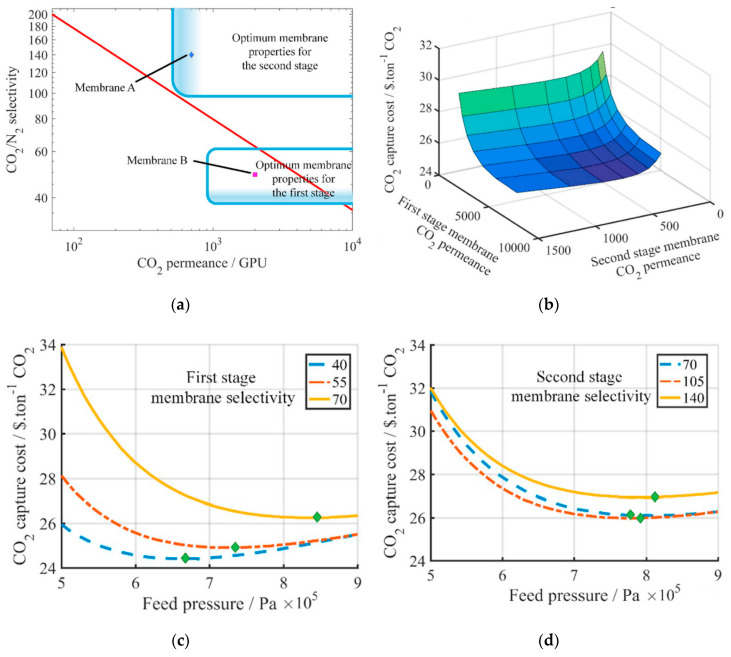
(**a**) Proposed scheme using two different types of membranes in Process E5 of Figure 4; (**b**) Effect of CO_2_ permeances of the two membrane stages on the capture cost (green = higher cost; blue = lower cost); (**c**) Effect of the first stage selectivity on the capture cost (second stage: 6 bar feed pressure, 140 selectivity); (**d**) Effect of the second stage selectivity on the capture cost (first stage: 6 bar feed pressure, 49 selectivity). Reproduced with permission from [34]. Copyright Elsevier, 2019.

The high selectivity requirement can be somewhat relaxed by using two different types of membranes in the two enriching stages. Xu et al. studied Process E5 in Figure 4 and proposed the use of a highly permeable but less selective membrane in the first enriching stage, and the use of a highly selective but less permeable membrane for the second enriching stage as shown in Figure 7a [34]. As discussed previously, the CO_2_ removal for the first stage needs to be as high as 90%, and the CO_2_ purity in the permeate is less than 50% regardless of the membrane selectivity. Therefore, a highly permeable membrane can be used in this stage for the bulk separation. In the second stage, a more selective membrane must be used in order to further purify the CO_2_ to >95%. 

Not surprisingly, the capture cost reduces with increasing CO_2_ permeances of both stages (Figure 7b). However, the optimal selectivity for the first stage is at 40 with a feed pressure of 6–7 atm (Figure 7c). Contrarily, the selectivity of the second stages should be increased to 105 and the optimal feed pressure also needs to be increased to ca. 8 atm (Figure 7d). Based on this assessment, they proposed the use of the Generation 2 Polaris™ membrane (Membrane B in Figure 7a: 2000 GPU, 50 selectivity [35]) developed by Membrane Technology and Research (MTR) for the first stage and an amine-containing facilitated transport membrane (Membrane A in Figure 7a: 700 GPU, 140 selectivity [36]) developed by The Ohio State University (OSU) for the second stage. 

This study indicates that the membrane performance for each stage in a multi-step process should be optimized individually due to the different feed compositions and stage cuts. In addition, only the feed compression was considered in this study, which concluded with relatively high feed pressures of 6–8 atm. A permeate vacuum should be considered at least for the second stage in order to further explore possibilities for a better process economics.

**Figure 8:**
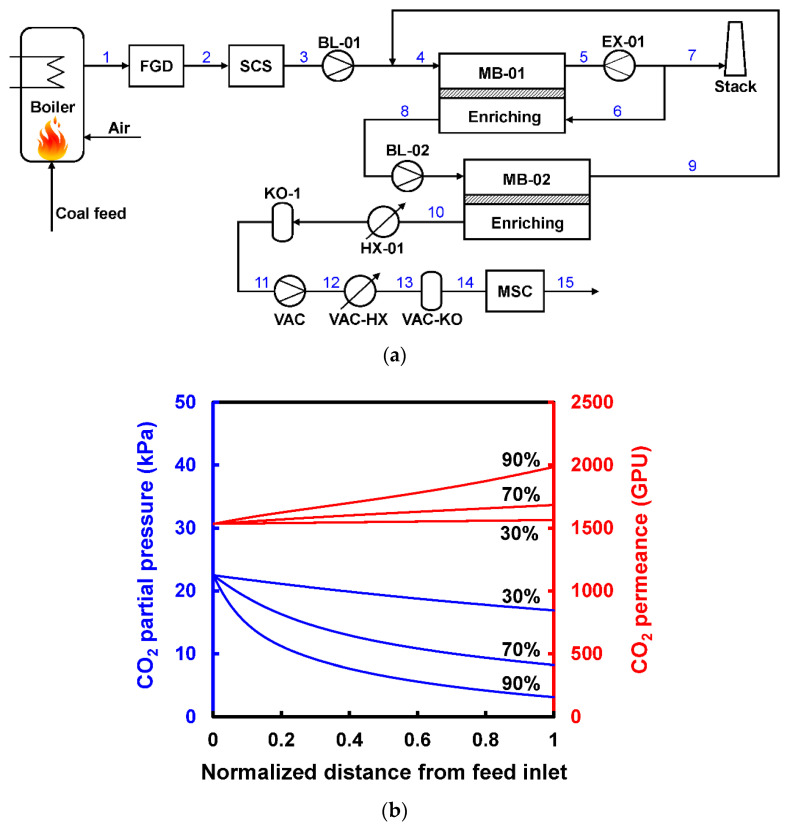
(**a**) Flow diagram of two-stage retentate recycle process to capture CO_2_ from coal-fired power plant; (**b**) Changes of CO_2_ partial pressure and the corresponding CO_2_ permeances in a facilitated-transport membrane module with 30%, 70%, and 90% CO_2_ recoveries. Reproduced with permission from [37]. Copyright ACS, 2020.

Another variation of the Enriching Cascade E5 is to recycle a portion of the CO_2_-lean retentate as an internal sweep gas [32,37]. This option is of particular interest for highly CO_2_-selective membranes since the sweep gas can provide an additional transmembrane driving force to fully utilize the highly selective feature. Han and Ho proposed a two-stage retentate recycle process as shown in Figure 8a, where 15% of the retentate (ca. 92% N_2_) of the first enriching stage is recycled back to the permeate side as a countercurrent sweep [37]. The retentate recycle enhances the CO_2_ permeation through the first enriching stage; therefore, the feed pressure can be reduced to ca. 3.5 atm compared to the 6–8 atm in the work by Xu et al. (see Figure 7c,d). More importantly, the N_2_-rich retentate recycle reduces the N_2_ permeation through the first enriching stage. This feature minimizes the N_2_ loss from the feed to the permeate side through the membrane, thereby more compression work is recovered by the retentate expander of stage one. 

Han and Ho and their coworkers also applied this design concept to an amine-containing facilitated transport membrane, in which the CO_2_ permeance increases with reducing CO_2_ partial pressure due to the mitigated carrier saturation phenomenon [38,39,40]. As shown in Figure 8b, the CO_2_ partial pressure reduces significantly upon the CO_2_ removal in the first membrane stage, especially at 90% CO_2_ recovery [37]. The reducing CO_2_ partial pressure leads to an uprising CO_2_ permeance along the feed flow direction. On the other hand, the N_2_ permeation is barely affected by the CO_2_ partial pressure since it depends on the solution-diffusion mechanism. Therefore, the separation becomes more efficient and selective owing to the mitigated carrier saturation. For the specific facilitated transport membrane studied, the membrane area can be reduced by ca. 12% if the carrier saturation phenomenon is considered.

**Figure 9:**
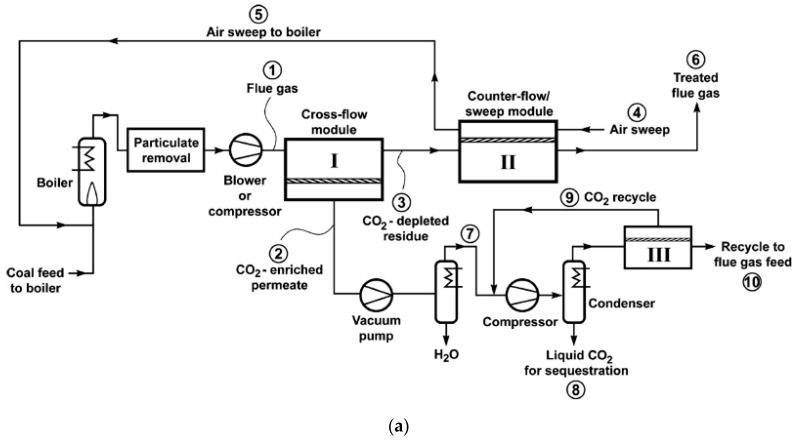

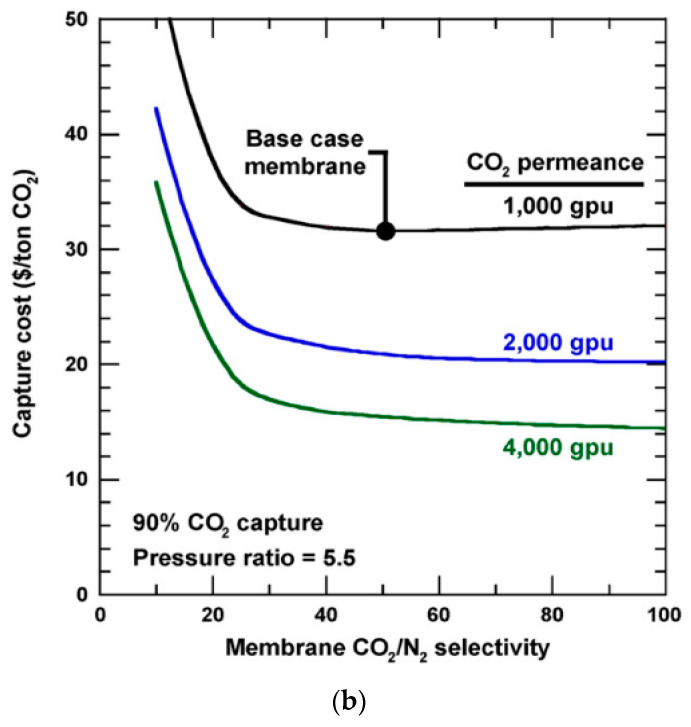
(**a**) Flow diagram of a two-step air sweep membrane process to capture and sequester CO_2_ from coal-fired power plant; (**b**) Effect of CO_2_/N_2_ selectivity on capture cost for 90% CO_2_ capture. Base case = 1000 GPU and 50 CO_2_/N_2_ selectivity. Reproduced with permission from [41]. Copyright Elsevier, 2010.

As discussed in Figure 4, the stripping cascade design is generally less cost-effective than the enriching cascades. By nature, it is challenging for the second stripping stage to produce high purity CO_2_ with a feed containing less CO_2_ than the flue gas. This issue can be partially addressed by Process S5 in Figure 4 via the permeate recycle. However, this option increases the feed flow rate to the first stripping stage, which inevitably requires a higher feed-to-permeate pressure ratio and thus a higher energy consumption. Merkel et al. from MTR integrated Process S5 with the boiler in a coal-fired power plant as shown in Figure 9a, in which the combustion air was used as the sweep gas for the second stripping stage [41]. The CO_2_-laden air was then fed to the boiler, which resulted in a higher CO_2_ concentration in the flue gas after combustion. The elevated CO_2_ concentration also provided a larger transmembrane driving force for the first stripping stage. The permeate of the first membrane stage was then further purified by cryogenic distillation to produce liquid CO_2_ with purity >95%. The air sweep eliminated the need for aggressive flue gas compression; the process could be operated with a feed at ambient pressure in conjunction with a permeate vacuum down to 0.2 atm for the first stage.

The cost sensitivity of the air sweep process was also conducted for MTR’s Generation 1 Polaris™ membrane (i.e., the base case membrane in Figure 9b) and hypothetical improved membranes with better permeances or selectivities [41]. As shown in Figure 9b, the CO_2_/N_2_ selectivity is deemed less important when it is above 50. Instead, the CO_2_ permeance is highlighted as the limiting factor for the capture cost, which stresses the need for highly permeable membranes with moderate CO_2_/N_2_ selectivity. The relaxed requirement for the selectivity is partially because of the use of cryogenic distillation. However, this energy-intensive operation adds onto the energy consumption and system complexity. 

Ramasubramanian et al. adapted this design concept but focused on highly CO_2_-selective membranes [42]. In order to eliminate the need of the cryogenic distillation and make the system cost-effective, a CO_2_/N_2_ selectivity greater than 140 is required, which is within the reach of a number of facilitated transport membranes. It should be noted that the selectivity of the air sweep membrane stage is less important than that of the first vacuum stage. In the various studies for this air sweep process, the process optimization with respect to membrane performance and operating pressures generally points to a 50% CO_2_ removal by the vacuum stage, resulting in a retentate containing 8–9% CO_2_ and 70–80% N_2_ [22,28,32,41,42,43]. Because the N_2_ concentration is close to that in air, the N_2_ flux is low regardless of the CO_2_/N_2_ selectivity. Therefore, the membrane for the air sweep stage can be less selective as long as it possesses sufficient CO_2_/O_2_ selectivity to minimize the O_2_ loss from the sweep air to the treated flue gas. Therefore, using two different types of membranes similar to those discussed in Figure 7 might be another opportunity to further improve the design of the air sweep process.

#### 2.2.3. Processes for Natural Gas-Derived Flue Gas 

The processes discussed in Section 2.2.1 and Section 2.2.2 all focus on the CO_2_ capture from coal-derived flue gases. Another important carbon-heavy source is the flue gas produced by a natural gas combined cycle (NGCC) power plant, where the high-temperature exhaust from the combustion turbine is passed to a heat recovery steam generator (HRSG) for generating steam and producing additional power by a steam turbine. Because of the excess air used in the combustion, the CO_2_ concentration in the flue gas is only 3–4%. Therefore, the carbon capture from a NGCC plant is more challenging than that from a coal-fired power plant.

**Figure 10:**
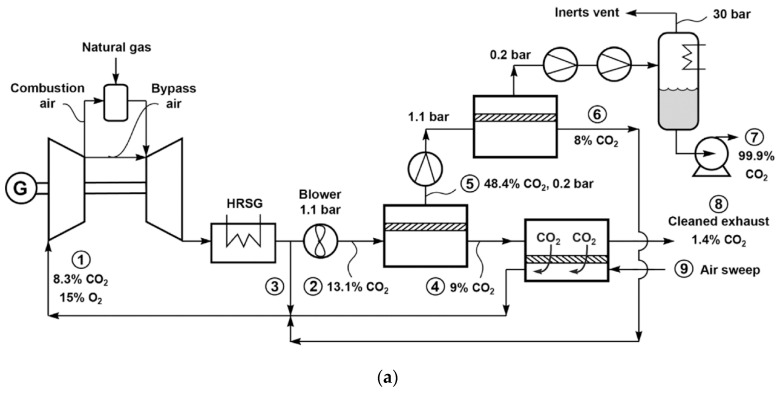

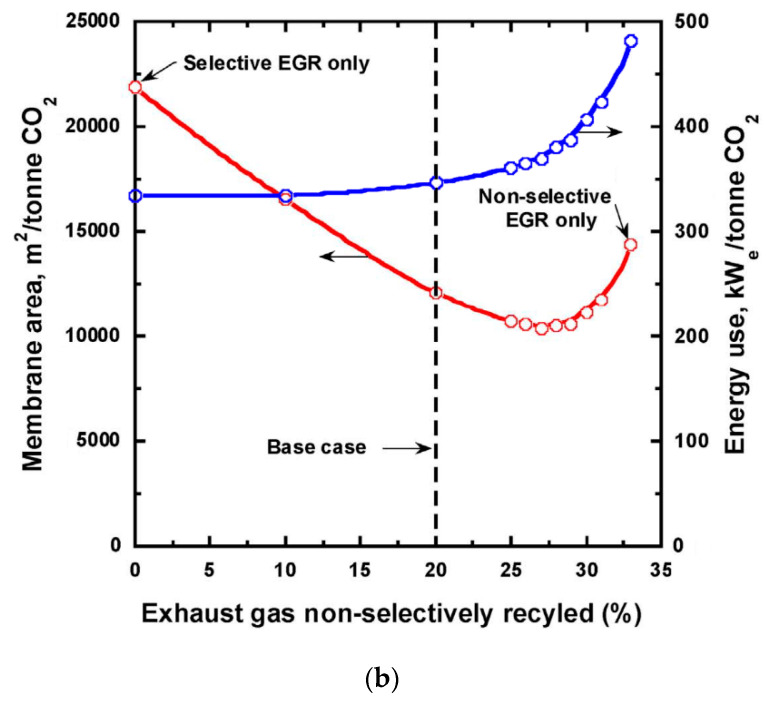
(**a**) Flow diagram of selective exhaust gas recycle (EGR) to capture and sequester CO_2_ from natural gas-fired power plant; (**b**) Effect of changing the non-selective EGR fraction on membrane area and energy use. Reproduced with permission from [45]. Copyright Elsevier, 2017.

In order to increase the separation driving force, a process so-called exhaust gas recycle (EGR) has been devised to recirculate a portion of the cooled flue gas after the HRSG (containing ca. 15% O_2_) back to the combustion turbine. Accordingly, the amount of fresh air is reduced and thereby, the CO_2_ concentration in the flue gas is increased [44]. Researchers from MTR adapted their combustion air sweep concept (see Figure 9a) to the EGR design, in which the carbon capture is integrated with the NGCC operation [14,45]. Figure 10a shows one of their designs [45]. As seen, a portion of the HRSG exhaust is directly recycled as the non-selective EGR. The remaining of the HRSG exhaust is treated by the two-step stripping cascade that is conceptually identical to that in Figure 9a, where the combustion air is used as the sweep for the second stripping stage. The CO_2_-laden air is fed to the combustion turbine, resulting in an additional selective EGR that helps increase the CO_2_ concentration to 13–21% for the membrane separation. 

Baker et al. studied different allocations of the non-selective and selective EGRs for a membrane with 2500 GPU CO_2_ permeance and 50 CO_2_/N_2_ selectivity [45]. As shown in Figure 10b, a greater extent of the selective EGR renders a lower energy consumption, while an appropriate degree of non-selective EGR can drastically reduce the membrane area. The optimal case appears to be the direct recycling of 20% of the HRSG exhaust with the remaining treated by the stripping cascade. They also studied the effect of membrane selectivity, concluding that a higher selectivity (e.g., a selectivity of 100) can significantly reduce the energy consumption, especially at 90% CO_2_ capture. 

A similar conclusion was arrived by Turi et al. [46], where a similar selective EGR process was studied for a facilitated transport membrane with a high CO_2_/N_2_ selectivity of 500 [47]. The better selectivity relaxes the feed compression requirement for the first stripping stage and leads to lower energy consumption. Also, using a more selective membrane eliminates the need of the auxiliary enriching membrane stage and the cryogenic distillation unit in Figure 10a, which makes the membrane-based process competitive to the MEA absorption for NGCC carbon capture. In contrast, van der Spek et al. concluded that the membrane-based process is not superior to the MEA absorption for a membrane selectivity of 50 [48].

**Figure 11:**
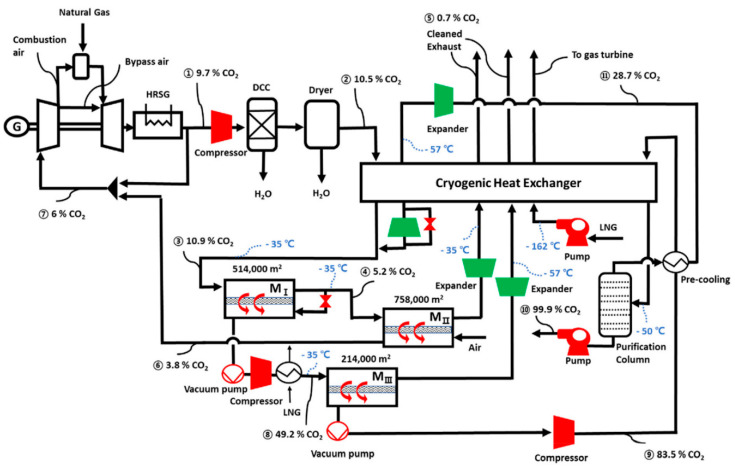
Flow diagram of the sub-ambient membrane process with selective and non-selective EGR for carbon capture from a natural gas combined cycle (NGCC) power plant. Reproduced with permission from [49]. Copyright Elsevier, 2020.

Another membrane based selective EGR process was proposed by Lee et al. by using a sub-ambient membrane [49]. As shown in Figure 11, the non-selective EGR section is the same as that in MTR’s process (Figure 10a). However, the rest of the HRSG exhaust is cooled to −35 °C through a heavily heat-integrated cryogenic heat exchanger, which is then passed to a three-stage membrane process for CO_2_ capture and selective EGR. For certain polyimide membranes, it is known that the CO_2_/N_2_ selectivity increases without a huge reduction on the CO_2_ permeance when operated at a sub-ambient temperature [50,51]. Therefore, a CO_2_/N_2_ selectivity as high as 100 can be expected. The membrane separation section is a combination of the retentate recycle process as shown in Figure 8a and the air sweep process as shown in Figure 9a. The retentate-recycle part is responsible for the CO_2_ removal and enrichment to >83% purity; the air sweep stage strips the remaining CO_2_ and recycles it to the combustion turbine. Their cost analysis indicates that both the capture cost and energy consumption reduce significantly with increasing membrane selectivity. Although the cryogenic process enables the high membrane selectivity and provides a CO_2_ enrichment factor ca. 1.2, other highly selective membranes at elevated temperatures might also be good candidates for this process.

## 3. Membrane Scale-Up, Modular Fabrication, and Field Tests

Although great progress has been witnessed in the past decade on the CO_2_ capture using membranes, most of the research remains at the beginning stage of the technology commercialization process. Noticeably, there are still challenges to scale up a membrane from laboratory to pilot scale, which is related to: (1) the limitation of the membrane separation performance (the trade-off between gas permeance and selectivity of most polymeric membranes) and (2) the membrane lifetime when exposed to the flue gas impurities such as SO_2_ and NO_x_. To the best of our knowledge, only a few research institutes or companies were able to advance their membrane technologies to the bench scale or above. These research advancements are discussed in this section. 

The pilot field trials include the Norwegian University of Science and Technology (NTNU) and SINTEF’s tests at the SINTEF Tiller plant (Trondheim, Norway) [52], the Norcem cement factory (Brevik, Norway) [53], the Colacem cement plant (Gubbio, Italy) [54,55], and the Sines bituminous coal power station (Sines, Portugal) [56]. Helmholtz-Zentrum Geesthacht (HZG) has also undertaken a field test at a hard coal power station (Baden-Württemberg, Germany) [57], while the Cooperative Research Centre for Greenhouse Gas Technologies (CO2CRC) and the University of Melbourne (UM) have conducted their membrane separation trial at a lignite-fired power station (Victoria, Australia) [58]. In South Korea, the Korea Research Institute of Chemical Technology (KRICT) has operated their pilot-scale membrane plant for the separation of CO_2_ from a liquefied natural gas (LNG) fired boiler (Daejeon, South Korea) [59]; Hanyang University (HYU) has also carried out the membrane module system testing in a pilot facility at the Korea Institute of Energy Research (KIER) [60]. In addition, MTR [35,61] and OSU [38,62] both tested their membranes in the coal-fired power plant at the National Carbon Capture Centre (NCCC, Wilsonville, AL, USA). The details of these tests are summarized in Table 1.

### 3.1. Plate-and-Frame Modules

Sandru et al. undertook a small pilot-scale plate-and-frame (PF) module testing at a power plant in Sines, Portugal, using real flue gas (12% of CO_2_, 6% of O_2_, ca. 600 mg/Nm^3^ of SO_2_, and 200 mg/Nm^3^ of NO_x_) for 6.5 months [56]. The effective area of the module was 1.5 m^2^, consisting of 24 pieces of polyvinylamine (PVAm) facilitated transport membrane sheets. During periods of continuous power plant operation, the membranes showed stable performances with CO_2_ permeances between 74 and 222 GPU and CO_2_/N_2_ selectivities between 80 and 300, which were similar to the values obtained in the laboratory at NTNU. Despite the harsh conditions such as power plant outages and high NO_x_ concentrations, the pilot testing still showed stable separation performances with a maximum of 75% CO_2_ in the permeate at a flow rate of 525 L/day.

**Figure 12:**
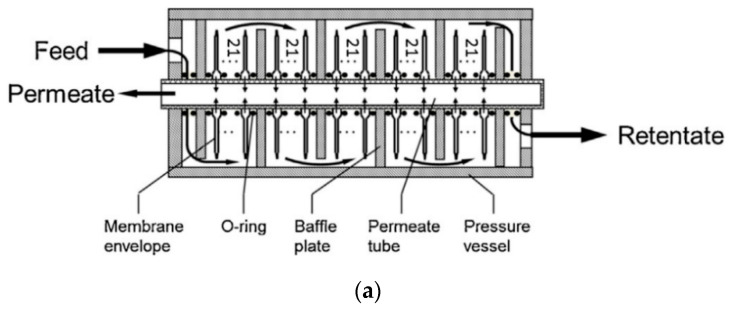

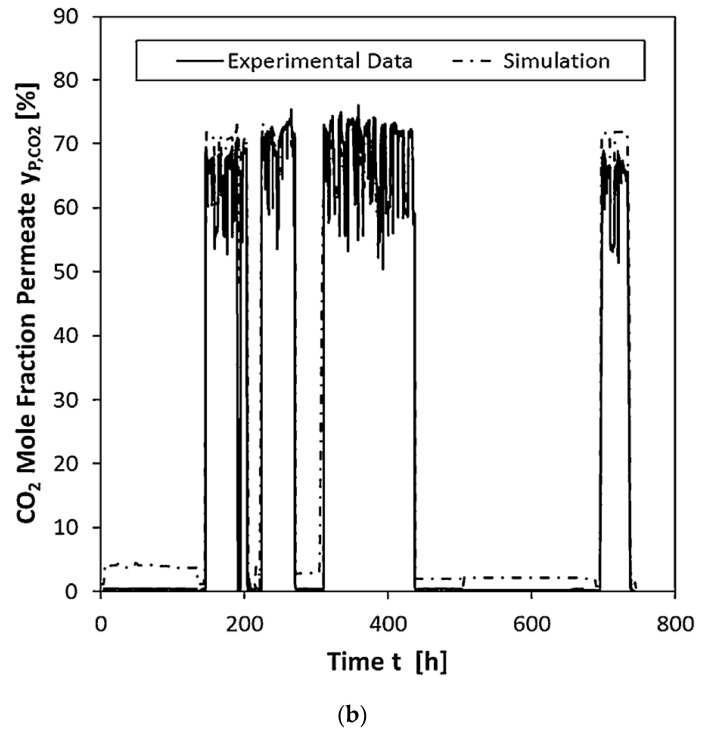
(**a**) Schematic representation of the 12.5 m^2^ PF membrane module fabricated by HZG; (**b**) CO_2_ purity on dry basis. Reproduced with permission from [57]. Copyright Elsevier, 2016.

The PolyActive™ membrane developed by HZG was also mounted into a pilot-scale PF module, which was installed in a hard coal-fired power plant, Rheinhafen-Dampfkraftwerk RDK-7 in Germany, to produce a CO_2_-enriched permeate stream for the cultivation of algae [57]. The effective membrane area of the module was 12.5 m^2^, and the schematic representation of the PF module is shown in Figure 12a. The flue gas from the power plant contained 14.5% CO_2_, 6.5% O_2_, 50–100 ppm of SO_2_, 76–91 ppm of NO_x_, and 14% of H_2_O with balance of N_2_. The feed and permeate pressures were 1.265 and 0.050 bar, respectively. A 740-h stable operation was achieved with a CO_2_ purity of 68.2% in the permeate and a recovery of 42.7% in a single-stage process (Figure 12b). The main highlight of this work was the effect of the pilot plant shutdown without subsequent air purging, which led to a decrease in the membrane separation performance. In order to avoid condensation on the membrane surface or in the associated rotating equipment, the flue gas needed to be pre-treated before entering the membrane module by removing dust, condensate, and most of the water vapor.

There are still some engineering challenges for the fabrication of the PF module. Aside from the well-known low membrane packing density [18], the PF modules are found to be difficult in upscaling. Yoo et al. has pointed out that defect control is the key for successful large-scale production without losing the intrinsic material properties [60]. In their work, Teflon™ AF2400, a commercial perfluoropolymer, was used as the protective layer material in thin-film composite (TFC) membranes. The prepared large-scale membranes were fabricated into PF modules. The whole module system included five PF modules connected in parallel, making the total effective membrane area about 5.7 m^2^. The membrane module system was tested in a pilot test facility at KIER located in Daejeon, South Korea. A CO_2_ purity of 74% with a CO_2_ recovery of 22% could be obtained, which proved that the protective layer method could be utilized in the production of large industrial-scale membranes to effectively control the defect formation.

### 3.2. Hollow-Fiber Modules

**Figure 13:**
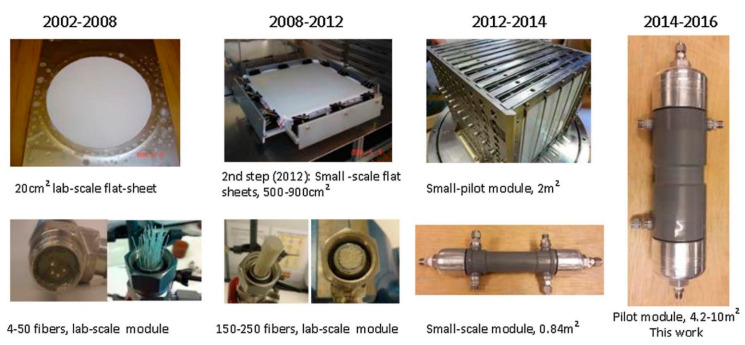
Upscaling history of NTNU’s flat-sheet and HF membrane modules. The rightmost photo shows a semi-commercial HF membrane module with 4.2-m^2^ membrane area. Reproduced with permission from [52]. Copyright Elsevier, 2017.

Besides the PF modules, the same PVAm-based facilitated transport membrane was also fabricated into hollow-fiber (HF) modules by NTNU for CO_2_ capture from the actual flue gas of a propane burner at the SINTEF Tiller plant, Trondheim, Norway [52] and the industrial gas from the Norcem cement factory, Brevik, Norway [53], respectively. He et al. demonstrated two semi-commercial HF modules coated with PVAm in-situ with a high packing density (i.e., membrane area of 8.4 m^2^), which performed in-parallel in a single-stage process [52]. Figure 13 exhibits the photograph of the 4.2-m^2^ semi-commercial HF module. The testing results indicated that a 60% CO_2_ purity was achieved in the permeate stream from a feed flue gas with 9.5% CO_2_ [52]. These researchers have pointed out that the key design parameters for the module (e.g., packing density and fiber dimension) should be well considered to achieve an optimized membrane module performance. The pressure ratio also needs to be taken into consideration because a lower pressure ratio (i.e., a lower feed pressure and/or a lower vacuum degree) is preferred from the perspective of energy consumption. However, a relatively larger feed-to-permeate pressure ratio (i.e., larger driving force) is also needed to give a higher CO_2_ flux and to reduce the required membrane area. Thus, it is important to balance these two factors via the tuning of the operating conditions.

In another study by NTNU, HF membrane modules containing up to 18 m^2^ of the same facilitated transport membrane was installed at the Norcem cement factory, Brevik, Norway for CO_2_ capture from an industrial gas containing 15–19% CO_2_ [53]. The pristine HF modules were received as commercial products from Air Products, Norway, and were coated with PVAm in-situ at NTNU. The testing results indicated a stable permeate with a CO_2_ purity of 65% over the accumulated 24 days via a single-stage process. The membrane also demonstrated a good stability even when exposed to high contents of SO_2_ and NO_x_ (100 and 5 ppm in average, respectively). This work intended to gain experience from the pilot testing and increase the technology readiness level (TRL) from level 5 to level 6. 

**Figure 14:**
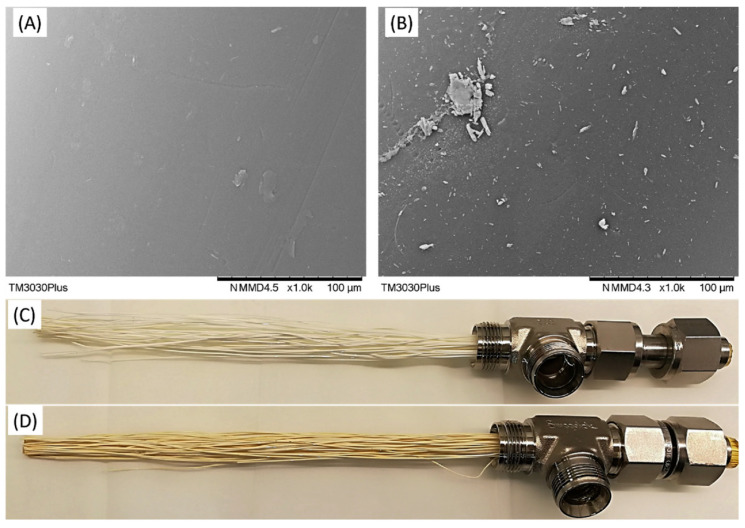
Comparison of different chemical-physical properties of the HF membrane modules before and after the field test at the Colacem cement plant: SEM images of the membrane surface before (**A**) and after (**B**) the field-test; Optical images of the hollow fibers before (**C**) and after (**D**) the field test. Reproduced with permission from [54]. Copyright Elsevier, 2019.

The facilitated transport membrane discussed above only relied on the PVAm, a fixed-site carrier, to achieve the CO_2_/N_2_ selectivity. Membranes containing amino acid salts as the mobile carriers have also been tested in the field. NTNU fabricated a facilitated transport membrane containing polyvinylalcohol (PVA) and an amino acid salt into small HF module with an area of 200 cm^2^, which was tested at the Colacem cement plant in Gubbio (PG), Italy [54]. A CO_2_ content of 50% in the permeate and a CO_2_ flux of 5 × 10^−3^ cm^3^ (STP) cm^−2^ s^−1^ were achieved at 90 °C. However, during the long-term stability test for a duration of one week, the CO_2_ flux reduced significantly to only around half of the original value. Presumably, the loss of the membrane performance was caused primarily by fouling. Severe membrane fouling occurred under humid conditions because of the suspended particulate matter as shown in Figure 14, in which Figure 14B clearly shows the particulate matter. The foulant could not be fully removed despite attempts of membrane regeneration, which might be the main cause of the reduced separation performance. This work highlighted the importance of flue gas pretreatment for the stable operation of the membrane. Besides, the occurrence of acidic water condensate caused by the 135–150 ppm of NO_x_ in the feed gas also generated potential damage to the membrane material. In addition, the high operating temperature (90 °C) and the high O_2_ concentration in the feed gas (11.5–14.0% CO_2_ and 12.5–14.5% O_2_) might potentially oxidize the reactive sites in the membrane.

Compared with the previous membrane containing the mobile carrier, new facilitated transport HF membranes were synthesized by NTNU by incorporating sterically hindered polyallylamine [55]. 200-cm^2^ HF modules were also tested at the Colacem cement plant. Although the permeances of the HF modules in the field seemed to be higher than those of the lab-scale ones, it was mainly due to the different test conditions. The researchers also compared HF modules with and without the mobile carriers. Those containing the mobile carriers clearly showed improved performances, indicating the advantages of the mobile carriers for post-combustion capture. The two-week testing of the HF module with untreated flue gas revealed a good durability of the membrane with a CO_2_ flux up to 750 NL m^−2^ h^−1^ and a CO_2_ permeate purity ranging from 50–55%. In addition, the presence of SO_x_ and NO_x_ (0–3 ppm of SO_2_ and 100–120 ppm of NO_x_) had a negligible effect on the membrane performance with the mobile carriers.

A pilot-scale membrane separation unit was tested with a LNG-fired boiler in the KRICT, South Korea, with a flue gas containing 10.8% of CO_2_, 2% of O_2_, and 87.2% of N_2_ [59]. The separation layer of the HF membrane modules was made of polyethersulfone (PES) with a CO_2_ permeance of 60 GPU and a CO_2_/N_2_ selectivity of 40. The HF modules were arranged in a four-stage enriching cascade, which achieved a CO_2_ purity of 99.2% and a recovery of 91.5% with feed and permeate pressures of ca. 5 and 0.2 atm, respectively. The experimental results were also compared with the results obtained from a process simulation. In some cases, the simulation showed different results, which, according to the authors’ declaration, were not fully understood. Also, further research is needed to investigate the effectiveness of the multi-stage membrane process to separate and recover CO_2_ from real emission sources by the energetic and economic analyses.

As a part of the Cooperative Research Centre for Greenhouse Gas Technologies (CO2CRC) H3 project, Scholes et al. investigated two different types of commercial membrane modules for CO_2_ capture from a lignite coal-fired power plant in Australia [58]. These two modules were: (1) the Air Products PRISM PA1020, which contained asymmetric polysulfone (PSf) hollow fibers, and (2) Dow Filmtec^®^ NF3838/30FF, a spiral-wound membrane module containing a polypiperazineamide with free amino groups. The results for the NF3838/30FF spiral-wound module will be discussed in the next section. The PRISM module with an effective membrane area of 5 m^2^ was operated for a total of 24 h. Considerable reductions in CO_2_ permeance (763 to 265 GPU) and selectivity (13 to 4) were observed after a few hours of membrane operation, which was explained by the plasticization of the membrane by water. The permeance and selectivity recovered slightly after some time of operation but still did not reach the initial values because of the permanently altered membrane structure caused by the humidity. Although proper flue gas pre-treatment could remove the water vapor, the overall low selectivity of the PRISM module made it unsuitable for the post-combustion capture of CO_2_.

### 3.3. Spiral-Wound Modules

As mentioned in the previous section, the NF3838/30FF spiral-wound (SW) membrane module (7.5 m^2^) was also tested at the lignite coal-fired power plant, which lasted for a total of 98 h [58]. Under the saturated water conditions, the CO_2_ permeance and CO_2_/N_2_ selectivity of the SW module increased, owing to the facilitated transport mechanism from the amino groups. However, both the CO_2_ permeance and CO_2_/N_2_ selectivity did not achieve the same level observed in the laboratory, possibly due to the competition from other acid gases, concentration polarization, and membrane fouling.

**Figure 15:**
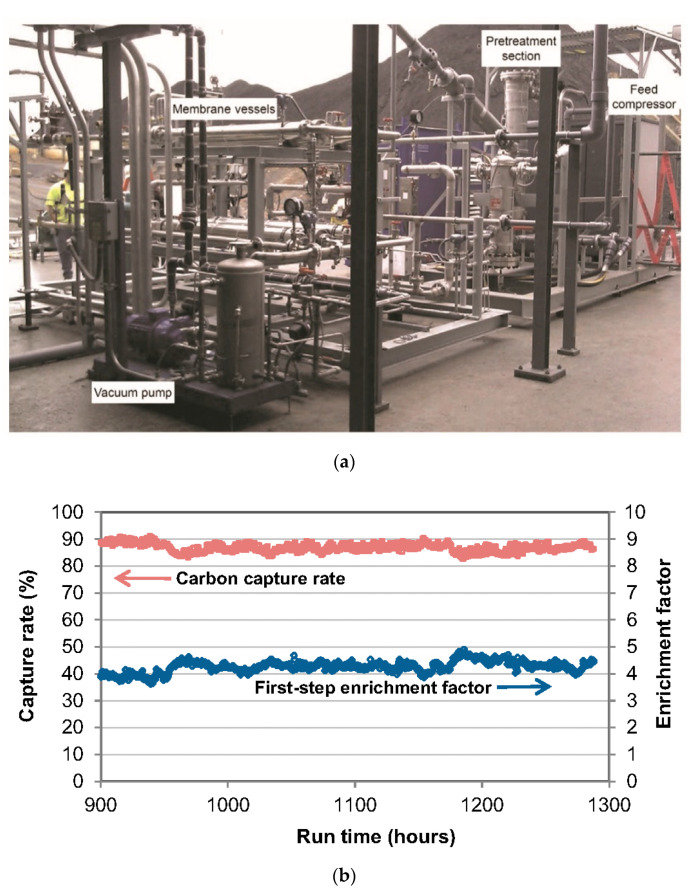
(**a**) Photo of MTR’s 1-TPD carbon capture system at NCCC; (**b**) Carbon capture rate and the enrichment factor of the first vacuum stripping stage. Reproduced with permission from [35]. Copyright Elsevier, 2015.

A more successful field test of SW modules was reported by MTR for their Polaris™ membranes [35,61]. In 2011, White et al. constructed a pilot-scale SW module system (Figure 15a) to capture CO_2_ from flue gas at NCCC in Wilsonville, Alabama, USA, which was sized to treat the flue gas at a capacity of 1 ton of CO_2_ per day (TPD) [35]. The SW modules used in this system were full-scale commercial ones with a diameter of 8 inches and a length of ca. 40 inches. As discussed in Section 2.2.2, MTR proposed a two-step stripping cascade with sweep air (see Figure 9a) to capture 90% CO_2_. The vacuum stripping stage and the air sweep stage, the two key elements in their process, were tested via the SW modules. The stability experiment was continuously operated for 1800 h with a capture rate of 90% (Figure 15b). In 2015, the CO_2_ capture capacity was expanded to 20 TPD [61]. The membrane system with 7 commercial SW modules was operated for over 1000 h with actual flue gas at NCCC, achieving a 90% CO_2_ capture rate in parametric testing and consistently capturing over 85% of the CO_2_ in the steady-state operation. Noticeably, MTR reported that their SW module was not suitable for the air-sweep stage due to the large pressure drop caused by the high sweep air flow rate. Therefore, new PF modules were designed and fabricated for the air-sweep stage in their 20-TPD skid, which demonstrated a pressure drop below 1 psi.

**Figure 16:**
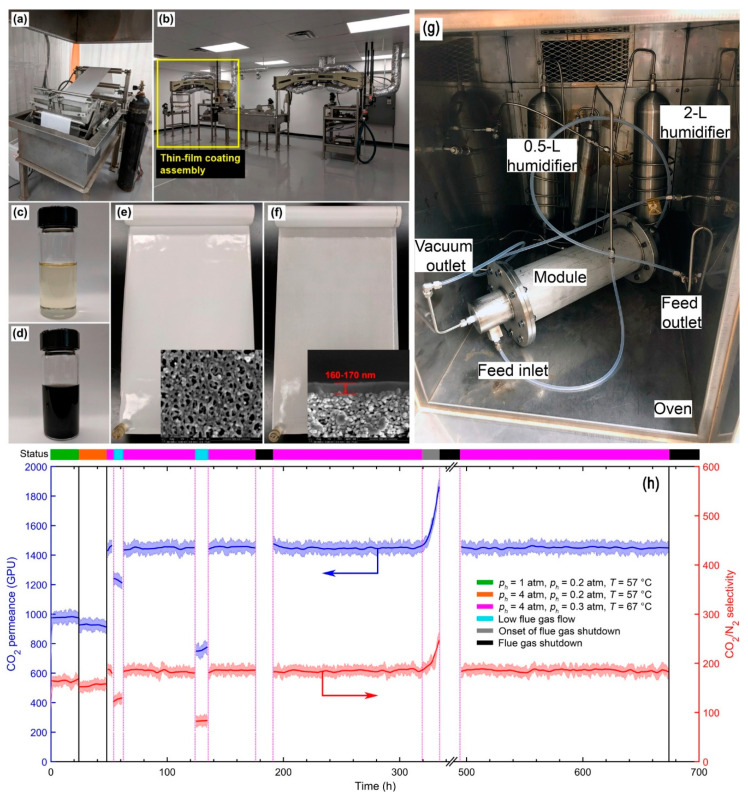
(**a**) Polymer support casting machine; (**b**) Thin-film coating machine; (**c**) PES casting solution for support fabrication; (**d**) Amine-containing coating solution; (**e**) 14″-wide scale-up PES substrate (insert: SEM of the PES surface morphology); (**f**) 14″-wide scale-up composite membrane (insert: cross-sectional SEM of the composite membrane’s top layer); (**g**) Photo of OSU’s 1.4-m^2^ SW module at NCCC; (**h**) 500-h stability of the SW module. Reproduced with permission from [38,63]. Copyright Elsevier, 2019&2020.

Another effort of the field test of SW modules was reported by OSU for their facilitated transport membranes containing both mobile and fixed-site amine carriers [36,38,62,63,64,65,66,67]. The roll-to-roll continuous membrane fabrication was demonstrated as shown in Figure 16a–f [63]. The 14″-wide prototype membrane was fabricated into a SW module with a membrane area of 1.4 m^2^, which was tested at NCCC with actual flue gas containing 2 ppm SO_2_, 1.5–4 ppm NO_2_, and 7.6% O_2_ (see Figure 16g) [38]. During the field trial, the SW module demonstrated a CO_2_ permeance of 1450 GPU and a CO_2_/N_2_ selectivity of 185 at 67 °C as shown in Figure 16h. A CO_2_ recovery of 44% was achieved by a single SW module with a CO_2_ purity of 94.5%. Overall, the module showed a 500-h stability despite various upsets due to flue gas flow rate variations and outages.

The stability and resilience of the facilitated transport membrane module have shed promising light on the following aspects. First, the amine carriers possessed essentially no volatility in the polymeric membrane, which eliminated the possibility of vaporization loss. Second, the low level of SO_2_ did not affect the transport performance of the carriers to a significant extent, which was likely due to the physical sorption of SO_2_ [68]; a cumulation of sulfur species in the membrane was not observed. Third, the chance of oxidation of the amine carriers at 67 °C was practically non-existent. Fourth, the polymer matrix was fully rubbery and not subject to a conformational relaxation, i.e., no physical aging. In addition, post-analysis of the tested membrane samples showed that no significant amounts of Cr, As and Se were deposited onto the membrane during the 500-h test at NCCC.

## 4. Conclusions

CO_2_ capture from coal- or natural gas-derived flue gas has been widely considered as the next opportunity for the large-scale deployment of gas separation membranes. Despite the advances in the synthesis of high-performance membrane materials, the modular fabrication of the membrane is rarely demonstrated in scale, and the membrane durability is seldomly tested with actual flue gas. In addition, the targeted CO_2_ recovery and purity of most membrane processes are yet to be verified in the field. The lack of experience in the field operation of a membrane system imposes the greatest challenge for its commercialization. Therefore, the recent progress in the engineering of polymeric membranes for post-combustion carbon capture has been reviewed in terms of capture system energetics, process synthesis, membrane scale-up, modular fabrication, and field tests. The key conclusions and remarks are as follows:

(1) The CO_2_ capture from a dilute source such as flue gas is intrinsically energy-intensive, showcasing low-energy consumption benefits of membrane process. The assignment of proper transmembrane driving force is the key to balance the second-law efficiency and the footprint of a membrane system.
(2) Limited by the membrane selectivity and practical feed-to-permeate pressure ratio, a single-stage membrane process can only partially capture the CO_2_. For a higher CO_2_ recovery, a multi-stage cascade design is mandatory.
(3) Enriching and stripping cascades are both suitable for 90% CO_2_ recovery, provided that sophisticated recycling streams are designed in the processes to enhance the CO_2_ flux. In order to achieve a >95% CO_2_ purity, a CO_2_/N_2_ selectivity greater than 50 is needed to make the process feasible. However, a higher selectivity (e.g., >100) is generally required for an optimized membrane-alone process.
(4) HF and SW modules are the preferred modular configurations due to their higher packing density and ease of manufacturing. Although less studied, PF modules also have applications in post-combustion carbon capture, especially in pre-pilot studies and situations where a high-pressure drop is unaffordable.
(5) The actual flue gases are invasive to the membrane operation. The frequently encountered challenges include the fouling by the particulate matter, the chemical degradation caused by SO_x_ and NO_x_, heavy metal deposition, and water-induced plasticization. These factors should be considered during the membrane development. In addition, flue gas pretreatment should be emphasized prior to a field trial.

## Figures and Tables

**Table 1:** Summary of field tests of membrane technologies.

Company/ Institute	Flue Gas Source	Location	Membrane * & Module ^†^ Types	Size	Duration	Purity	Recovery
SINTEF [56]	Coal	Portugal	FTM; PF	1.5 m^2^	6.5 months	75%	N/A
HZG [57]	Coal	Germany	PolyActive™; PF	12.5 m^2^	740 h	68.2%	42.7%
HYU [60]	N/A	Korea	MMM; PF	5.67 m^2^	N/A	74%	22%
NTNU [52]	Propane	Norway	FTM; HF	4.2 m^2^	N/A	60%	N/A
NTNU [53]	Cement	Norway	FTM; HF	18 m^2^	24 days	65%	N/A
NTNU [54]	Cement	Italy	FTM; HF	200 cm^2^	1 week	50%	N/A
NTNU [55]	Cement	Italy	FTM; HF	200 cm^2^	2 weeks	50–55%	N/A
KRICT [59]	LNG	Korea	PES; HF	N/A	N/A	99.2%	91.5%
UM [58]	Coal	Australia	PSf; HF	5 m^2^	24 h	N/A	N/A
UM [58]	Coal	Australia	Polyamide; SW	7.5 m^2^	98 h	N/A	N/A
MTR [35]	Coal	USA	Polaris™; SW	1 TPD ^‡^	1800 h	N/A	N/A
MTR [61]	Coal	USA	Polaris™; SW&PF	20 TPD	1000 h	N/A	N/A
OSU [38]	Coal	USA	FTM; SW	1.4 m^2^	500 h	94.50%	44%

* Membrane type: FTM = facilitated transport membrane; MMM = mixed matrix membrane; PES = polyethersulfone; PSf = polysulfone. ^†^ Module type: PF = plate-and-frame; HF = hollow-fiber; SW = spiral-wound. ^‡^ TPD = ton of CO_2_ per day.
